# Service users’ perspectives in the design of an online tool for assisted self-help in mental health: a case study of implications

**DOI:** 10.1186/1752-4458-8-2

**Published:** 2014-01-09

**Authors:** Deede Gammon, Monica Strand, Lillian Sofie Eng

**Affiliations:** 1 Norwegian Center for Integrated Care and Telemedicine, University Hospital of North-Norway, Tromsø, Norway; 2 Center for Shared Decision-Making and Collaboration Research, Oslo University Hospital, Oslo, Norway; 3 Department of Psychiatry Blakstad, Division of Mental Health and Addiction, Vestre Viken Hospital Trust, Asker, Norway

**Keywords:** Service user involvement, Community-based participatory research, Patient involvement, Community mental health, Internet, Assisted self-help, Mental health systems

## Abstract

**Background:**

The involvement of persons with lived experiences of mental illness and service use is increasingly viewed as key to improving the relevance and utility of mental health research and service innovation. Guided by the principles of Community-Based Participatory Research we developed an online tool for assisted self-help in mental health. The resulting tool, PsyConnect, is ready for testing in two communities starting 2014. This case study reports from the design phase which entailed clarifying very basic questions: Who is the primary target group? What are the aims? What functions are priorities? Roles and responsibilities? What types of evidence can legitimize tool design decisions? Here we highlight the views of service users as a basis for discussing implications of user involvement for service design and research.

**Case description:**

PsyConnect has become a tool for those who expect to need assistance over long periods of time regardless of their specific condition(s). The aim is to support service users in gaining greater overview and control, legitimacy, and sense of continuity in relationships. It has a personalized “my control panel” which depicts status → process → goals. Functionality includes support for: mapping life domains; medication overview; crisis management; coping exercises; secure messaging; and social support. While the types of evidence that can legitimize design decisions are scattered and indirectly relevant, recent trends in recovery research will be used to guide further refinements.

**Discussion:**

PsyConnect has undoubtedly become something other than it would have been without careful attention to the views of service users. The tool invites a proactive approach that is likely to challenge treatment cultures that are reactive, disorder-focused and consultation-based. Service user representatives will need to play central roles in training peers and clinicians in order to increase the likelihood of tool usage in line with intentions. Similarly, their influence on tool design has implications for choice of methods for evaluation.

**Conclusions:**

Starting down the path of service user involvement in intervention design fosters commitment to follow through in the remaining implementation and research phases. While this can be time-consuming and less meriting for researchers, it is probably vital to increasing the likelihood of success of person-centered service innovations.

## Background

The involvement of persons with lived experiences of mental illness and service use is increasingly viewed as key to improving the relevance and utility of mental health research and service innovation [1,2]. Indeed, funding bodies increasingly require user involvement as a prerequisite for awarding grants [3]. One approach to user involvement is community-based participatory research (CBPR), which is characterized by systematic inquiry, with the participation of those affected by the health problem, for the purposes of education and action or effecting social change [4]. Rather than being a research method, CBPR is an approach towards research that emphasizes “equitable” engagement of all stakeholders throughout the research process, from problem definition through data collection and analysis to the dissemination and use of findings to help effect change [5]. CBPR begins with a topic of importance to the community with the aim of combining knowledge and action for social change to improve community health and eliminate health disparities [5].

The current study started with the question of how information and communication technologies (ICT) such as Internet and mobile phones can improve user involvement and collaboration in community mental health services. Evidence of the effectiveness of online mental health tools is accumulating rapidly, along with knowledge of how to maximize benefits [6,7]. Nevertheless, it typically takes 14–17 years before this type of knowledge is translated into practice [8]. The speed with which Internet and mobile technologies emerge and are replaced outstrips our knowledge of their effects, and our ability to exploit them in healthcare. While controlled studies are essential to our knowledge base, equal attention is needed on refining research designs that iteratively incorporate experiential and research-based knowledge into design and evaluation processes in real life contexts [9]. This is vital in ensuring that results are timely, relevant and beneficial to those who we seek to help.

This case report is from a 1½ year pre-project and development phase of an online tool for assisted self-help in community mental health. Guided by the principles of CBPR we developed a tool called PsyConnect, which will be tested from the start of 2014 in two “communities” – i.e. service users and their service providers within primary and specialist levels of care in the north and south of Norway. The design specifications resulting from these processes serve as a point of departure for this paper. We retrospectively reconstruct the processes and rationales that led to answers to the overriding questions that stakeholders initially faced: Who is our primary target group? What objectives and outcomes should we have? What functions and content should be prioritized? Who is responsible for appropriate use of the tool? What types of evidence can legitimize our decisions?

This case report highlights the perspectives of service users in this process. The objective is to illuminate the implications of service user involvement in service innovation and research, thus serving as a basis for discussion about how service user involvement might further evolve in light of new technologies for assisted self-help.

## Case description

### Starting with an idea and a question to community stakeholders

In line with CBPR, researchers contacted stakeholders within two Norwegian regional community service centers for mental health with an unrefined idea: to adapt an existing online tool for assisted self-care to the needs of service users in mental health. Stakeholders included the local Learning and Coping Center, which trained many service users in how to contribute their experiential knowledge, e.g. in projects, committees and teaching. Other stakeholders included a hospital and its affiliated outpatient clinics, and local municipal units that provide all primary health and care services. Through a series of meetings, researchers gave an overview of available online health tools for assisted self-help and presented research supporting their potential for improved care [10-12]. One of these tools was Connect 2.0, a platform that has been extensively tested for cancer patients, and that includes several modules: a) secure messaging, b) discussion forum, c) symptom-registration which interacts with d) symptom-specific self-help information, and e) a diary [13,14].

Stakeholders were asked: *Can community mental health service users and their providers benefit from a tool like this? If so, will you participate in determining how it should be adapted to best meet the needs of service users?*

All stakeholders confirmed their commitment to this endeavor under the condition of funding. Funding the work required to prepare grant proposals that will in turn secure funds for the actual project can be a stumbling block for CBPR [15]. Fortunately, several stakeholders, including service users, volunteered their time in a pre-project that included writing a grant proposal, and funding was awarded from the start of 2012. This in itself was a milestone since CBPR protocols diverge from traditional health research in ways that can make them difficult to fund [16]. Thus, we embarked on a process with no specific goals other than to design a system that stakeholders could believe in and commit to testing.

#### Practice-research team

Once funding was secured, we established a practice-research team in line with the principles of CBPR [17]. The team includes three service users, representing a total of 45 years’ first-hand experience of mental health care for various mental health conditions. Their ages ranged from 25 to 50 years. These team members committed to contributing as much of their time and knowledge as they were capable. One service user (LSE) was employed 80% to work more consistently with the programmers and to ensure continuous input from the other service users. Other team members included clinicians from the municipality, outpatient clinic and mental health hospital, together with IT-experts who participated as required. We also worked to build a broader network through open workshops in the north and south of Norway. DG functioned as team leader and PhD candidate MS was coordinator.

#### Mutual learning and trust

Monthly meetings in the practice-research team lasted for 4 hours with a break for lunch. The first few meetings focused on getting to know each others’ experiences, capabilities and interests in the project, and on discussions of our overriding questions. We continuously worked to create an environment of trust where no question or issue was too big or small. Service users learned to help researchers and clinicians tame their jargon, often using humor. Participants were encouraged to suggest items for the next meetings’ agenda, and continuous dialogue by phone and email between meetings was also encouraged. Educational sessions (e.g. available internet tools, therapeutic approaches) were also scheduled as part of several meetings. The monthly meetings were documented with minutes detailing discussion topics, decisions and plans. Slowly but surely we became a well-functioning team that shared a common understanding of what we wanted to achieve and how.

### Overriding questions

The sections below are organized around the overriding questions that guided discussions throughout the process: Who is our primary target group? What objectives and outcomes should we have? What functions and content should be prioritized? Who is responsible for appropriate use of the tool? What types of evidence can legitimize our decisions? Here we summarize team discussions and the rationales for decisions made, highlighting the views of service users.

#### Who is our primary target group?

Who should we work to help? Discussions of this topic touched on various dimensions such as needs during different phases (preventative/pre-diagnosis, treatment, rehabilitation), diagnoses (e.g. affective, psychosis), as well as service issues (e.g. fragmentation, resources). Rather than “copying” existing, well-tested diagnosis-specific tools for mild conditions (e.g. anxiety, depression, PTSD) – which we assumed that we could adapt if appropriate – we decided to address the largely unmet needs of those with persistent conditions and multiple service providers. Increasingly, service users held that our work should be *non-condition-specific* and *person-centered* and support continuity across levels of care (primary, secondary, tertiary). The following paraphrased quote from service users on the team illuminates the rationale for this focus:

“I’ve had numerous diagnoses over the years, and none of them seem to influence the treatment I receive. I want a tool that I can use over time, independent of my current condition, or the favorite theory of whoever happens to be my main provider at a given time”.

While this person-centered perspective became a guiding premise in the design process, it also introduced concerns, particularly in light of local and national efforts to disseminate best practice protocols. Clinicians and researchers were concerned that the broader and less specified the target group, the greater the difficulty of gaining legitimacy among clinicians, as well as in detecting (publishable) outcomes. Nevertheless, the challenges of those with multiple conditions in need of long-term support in everyday life evolved as our primary focus.

#### What are our objectives and expected outcomes?

Researchers and clinicians tended to focus on goals reflecting traditional outcomes such as symptom reduction, adherence, service consumption, and satisfaction. While this was acceptable to service users, they argued that goals formulated along these lines were uninspiring and little related to their lives. After numerous discussions around this issue we ultimately arrived at the following common vision: *PsyConnect supports service users in guiding their lives in the direction they choose, in accordance with their personal values.* This was broken down into three main goals for our work. “PsyConnect aims to support mental health service users in:

1) *Gaining an overview and greater control over aspects of their personal lives that affect their health and well-being.*

2) *Legitimizing their personal knowledge, strengths and values in the formation of services provided by healthcare.*

3) *Experiencing a greater sense of continuity of care and relationships with and between providers”.*

Service user representatives held that these goals were key for achieving the more traditional outcomes sought by clinicians and researchers. In addition to avoidance of diagnosis-driven content, service users held that the tool should reflect generic, “humanistic” needs and values. Thus, we decided to try for both: a flexible, generic support tool that also allowed for subsequently incorporating other, more condition-specific protocols as appropriate.

#### What functions and content should be prioritized?

The functions that we ultimately decided upon as key to achieving the above objectives are available to service users through a secure user interface that conforms to Norway’s strict data security standards [18]. The specific functions are listed below in Table 1 in the left-hand column. The right-hand column summarizes the rationales discussed within the team, particularly highlighting the views of service users.

**Table 1:** Functions in the service user interface and rationales for design

**Functions as presented to service users in PsyConnect**	**Main rationales from the team, with an emphasis on service user perspectives**
**What is important in my life:**	
Here you can write a brief statement about your values, or the things that matter most to you in your life.	Although several team members found the concept of values difficult pin down, they were inspired by literature that addressed values, or similar concepts such as purpose, meaning, hope. The value statement is visible on the opening page.
**Life domains:**	
Here you can describe your current situation within different life domains (school/occupation, social life, mental and physical health, housing, finances). You can also describe what helps and hinders you in living as you would like.	This module departs from the original symptom-monitoring module found successful for cancer patients [13]. Our service users argued that symptom monitoring would be unhelpful and “depressing” in bad periods. Instead, they held that free text options for describing their status in different life domains would not only help them gain better overview, but also facilitate constructive communication with providers.
**My medications:**	
Here you can list which prescribed and non-prescribed medicines you take, how they are intended to help you, your experiences with them, and notes or questions to your doctor.	Services users report uncertainties about their various medications, both prescribed and non-prescribed. Also, they often forget to broach their questions and concerns during consultations. The information in this module is accessible to providers only when the service user sends it as a message. (See next section about responsibilities.)
**Network map:**	
Here you can make a map of all the people you have a relationship with. You can change their placement on the map according to how close or distant a relationship you feel.	Service users have found paper drawings of networks to be valuable, particularly during lonely down periods. In PsyConnect, contact information is linked to each respective person in the map.
**Exercises:**	
Here you will find different exercises that can help you strengthen your skills in areas that you might want to improve. The exercise categories are: coping, strengths, collaboration, and lifestyle. You can also make your own exercises, and ask to be reminded to do exercises according to your own schedule.	Service users find that having exercises to do between consultations is a good way to maintain focus. The categories and exercises mainly focus on building resources, although some support problem analysis. Services users and their providers select exercises and can design new exercises according to needs.
**Crisis management:**	
Here you can make an overview of warning signs or triggers and plan how you can prevent yourself from getting worse. You can also specify what you want your helpers to do if you experience an acute situation.	This is an online version of a paper-based form for crisis management that team members had positive experiences with. Service users believe that having the crisis management plan available online will make it easier for them to make relevant updates and, importantly, automatically update their helpers.
**Monitoring:**	
Here you can choose to register information about various aspects of your daily life, for example, sleep, nutrition, physical activity, social life, medications, and assessment of relations with helpers. If you make registrations over a period, you can create a graph to help you see how these aspects might be related to your health and well-being.	While researchers sought standardized registrations, service users wanted a simple selection of smiley-icons that depicted their mood at a given time, along with a free-text field for notes. Also, they view the incorporation of interactive lifestyle-related content to be an important dimension for mental health.
**Goals and activities:**	
Here you can formulate goals that you want to work towards. You can also describe activities that can help you achieve these goals. If you want helpers to assist you, you can invite them to participate.	Service users have had good experiences formulating and working with goals and maintained that this was an important function. They wanted goals to be displayed as processes, organized thus: status (e.g. life domains) → process (e.g. activities, exercises) → goals.
**Good to know:**	
Here you will find information about PsyConnect and how you can adapt it to your own needs and daily life. You will also find a list of links to articles and information about mental health and well-being. Short stories and articles from other service users are also found here.	It is important that the workspaces are not overloaded with information, although much is available through “read more” links. All available information has been assessed for quality and all sources are cited.
**My helpers:**	
Here you can list contact information to your helpers and family.	This information is available to all those with access to the service user’s site. Service users decided that family members would only have access if service users allowed them to use service users’ personal ID.
**Messages:**	
Here you can communicate with your helpers in a secure way, and the content can be integrated with providers’ various electronic medical records.	This module was barely discussed as it is fundamental to the collaborative profile of the tool.
**Forum:**	
In the discussion forum you can anonymously meet other users of PsyConnect. There you can share experiences with others in similar life situations.	To underline the social support profile of this forum, it is only available to service users, although it is monitored by a health professional (who is not acquainted with the users).
**Diary:**	
This is your personal notebook where you can jot down thoughts, memories, or ideas. This might be useful in preparation for consultations, or for follow-up questions.	There were few discussions about this module since it was already available and believed useful by service users. It is not accessible to anyone but the user.

Many of the functions are displayed within a “my control panel” image that gives a sense of direction from status (e.g. life domains) → process (e.g. activities, exercises) → goals. One of the most important advantages service users expected of PsyConnect is that they would no longer need to repeat the same information over and over again for new providers. They envision this contributing to a sense of continuity for both themselves and providers.

#### Who is responsible for appropriate use of the tool?

Issues related to responsibilities arose throughout the design processes. Our assessment of similar tools [10,19,20] found that they were mainly used at clinics either from pre-consultation kiosks and/or during consultations. To our knowledge, the information generated during these encounters remains accessible to service users only when they are together with their clinician, who is responsible for quality and data protection.

In contrast, we sought a tool that could serve as a shared “meeting place” where service users can work either independently or together with providers, who can also access the site independently. Shared access – key to the collaborative profile of the tool envisioned by our team – nevertheless poses challenges. In particular, clinicians expressed concerns about the legal status of user-generated content and whether they would be held responsible for faulty or alarming content. Service users have held that the tool should be “owned” by service users. Thus, service users should also be responsible for the information that they put into the tool. As one expressed it:

“We are ultimately responsible for our own recovery process in any case, why not also for PsyConnect?”

Despite underlining both on the website and in individual written agreements that service users were responsible for content and use, clinicians remained uneasy about having access to service users’ sites. This was particularly the case for the medication module. Rather than having access, they preferred to receive information/questions directly from service users in the form of a message. If clinicians judge information in PsyConnect relevant to the medical record, they can transfer it to medical records through standard national message formats without having to log onto the tool separately.

Services users, on the other hand, reacted to these types of efforts to “protect” providers (e.g. legally and practically). They argued that if it is in fact best for service users that providers use the system in specific ways, then providers should be willing to do so. Regarding family members’ access, service users decided that they should only have access if service users themselves allowed family members to use service users’ personal ID. The pilot study will help illuminate these issues. Concerning our efforts to anticipate and assess risks, and formulate appropriate, clear-cut legal guidelines, our legal advisor noted that it is not possible to avoid all potentially unwanted situations through regulations; nor should regulations substitute for relational trust and good communication.

#### What types of evidence can legitimize our decisions?

Listening to and comprehending service users’ and clinicians’ insights and priorities, while coupling this with research that helped us critique and refine our design decisions, was a continuous, iterative process. While the evidence-base of diagnosis-specific interventions designed to reduced symptoms is substantial, research supporting the type of generic, multiple-module, user-controlled tool that was taking shape is scattered and only indirectly relevant.

During the early stages of this process, various domains of research were examined in terms of their ability to justify and elucidate the objectives and priorities of our team. Among these are approaches loosely referred to as “Third wave psychotherapies” which are gaining empirical support [21,22]. These are a heterogeneous collection of approaches, all of which build on the principles of cognitive behavioral therapy (CBT), but which are modified by approaches such as acceptance and commitment treatment, mindfulness, and behavioral activation. These can in turn be linked to transdiagnostic approaches to emotional disorders, approaches that have emerged in light of the high rates of current and lifetime comorbidity, and the cross-over effects of treatments based on CBT [23]. Several of the exercises in our database are derived from these approaches, although we have avoided flagging any specific approach.

Another domain of research – self-management in chronic care – can be said to take the “transdiagnostic” perspective a step further by distilling common principles of care across mental and physical disorders [11,24,25]. A number of well-tested guidelines for self-management across conditions have emerged from this literature and have inspired some of the content in modules related to physical and mental coping strategies. We nevertheless found that much of this literature was more medically oriented than was appropriate for our purposes.

The domain that ultimately resonated closest with the team’s values and objectives stems from recent trends in recovery literature [26,27]. In March 2012, the Substance Abuse and Mental Health Services Administration (USA) announced an updated working definition of recovery as: “a process of change through which individuals improve their health and wellness, live a self-directed life, and strive to reach their full potential” [28], p. [3]. Notably, this contrasts with traditional medical models of mental illness where “clinical” or “service-based” definitions of recovery are equated with cure [26]. Rather than recovery *from* mental illness, which implies cure as the goal, this new approach focuses on supporting the process of being *in* recovery. The team found that much of what we had arrived at during the design process reflected this orientation to recovery and that further revisions of the tool should seek to harmonize even closer to the domain of recovery.

Researchers’ concern with finding legitimacy for design decisions, and in choosing validated evaluation instruments, gave rise to crucial discussions within the team. Service users reported being “fed-up” with some of the standardized instruments which the researchers were considering. They found the instruments invasive and irrelevant to their lives: that the instruments failed to reflect our goals of overview/control, legitimacy and continuity. The publication of a systematic review of instruments for measuring mental health recovery [26] was timely in helping us narrow down our search for instruments. Importantly, an inclusion criterion for instruments assessed in this review was that users had been involved in their development. Since none of these are in Norwegian, we will initiate translation and validation of the instruments selected by our team, simultaneously refining new measures proposed by our service users.

#### The next steps

The next phase of the project will pilot test PsyConnect in two communities from the start of 2014. We will continue to iteratively adapt the tool together with local stakeholders in a small rural community in northern Norway, and a large urban community in southern Norway. Communities include primary, secondary and tertiary levels of care that are involved in the care of the recruited service users. The practice-research team that was responsible for development will be reorganized into two local teams with respective steering committees that will oversee the piloting processes. These committees will also include service users that have not been involved in the development phase. The timeframe for the pilot study is one year.

## Discussion and evaluation

PsyConnect has undoubtedly become something other than it would have been without significant influence from service users. It has resulted in a tool that service users on our team find relevant for their needs in daily life, but that also challenges current practices in mental health care. While clinicians’ wishes and concerns have been addressed, PsyConnect is a service users’ tool, and it represents a shift in activity and locus of control towards service users. After wandering among many different types of evidence to legitimize our decisions, we have found a “home” in more recent approaches to recovery, where evidence remains to be amassed [26]. We hope that PsyConnect can eventually serve as a platform for collecting evidence and further developing this approach.

Outsiders have periodically questioned the representativeness of the service users and clinicians on our team. No one has questioned the representativeness of the researchers. Obviously, the representativeness of participants will be an important issue after the pilot, when we test the tool under controlled conditions. For innovative discovery processes such as ours, shared visions and values, along with breadth of experience and roles among team members, are fundamental. The process of design has been genuinely co-creative. Distinguishing between respective contributions, as done in this paper, somewhat obscures the dynamics of what took place. Team members’ knowledge and opinions evolved over time, and were driven towards common goals by mutual curiosity and respect for diverse areas of expertise.

While PsyConnect can facilitate recovery-oriented practices, it is only a tool. Its value will be closely linked to organizational and cultural adaptations within clinical practices that are aligned with recovery thinking. Our main concern is that clinicians will miss opportunities to proactively engage service users in their own recovery. This will not be due to unwillingness, but rather to a clinical culture that is more consultation-based and reactive, than proactive. It is well known that adherence to, and positive outcomes of, online self-help tools increases significantly when coupled with prompts like “how did you do on your last exercise?”, even if these prompts are automatically generated [29]. While our tool also allows for automated prompts, it is mostly designed to strengthen existing relationships between service users and care providers within their community. Our experience suggests that this will be easier to achieve within the municipal health services, than within specialist services.

Clinicians’ concerns about being swamped with messages and having to reorganize their schedules to formulate responses can be an important barrier. We have not found any evidence to support these concerns, although this issue will be followed closely during the pilot. Rather, we hypothesize that the majority of service users will not misuse the lowered threshold for contact through PsyConnect, but will rather be respectful of clinicians’ time constraints.

We have little knowledge about levels of digital access and literacy within our target group. Internet penetration in Norwegian households is currently 94% [30]. Once national data security regulations for mobile health applications are in place next year, we will move PsyConnect to a mobile phone-based platform, which we assume is more accessible to our group. The pilot community in the North has already noted that several interested PsyConnect users lack PCs and Internet access. Thus local initiatives are underway to find used PCs and fund training. As quality-assured online health interventions grow in number, disparities in Internet access will become unacceptable in countries like Norway, where equity in access to healthcare, regardless of income or geography, is a fundamental right.

A major insight from the processes described in this case report is that service user involvement – to the extent that we have practiced it – obligates. This is not just due to personal relations, or to our commitment to following a CBPR approach. It unfolds from what PsyConnect has become. No one is more capable in conveying the intentions and functionalities of PsyConnect than the service users on our team and their network of peers. Their role in ensuring the quality of training of service users and clinicians in the pilot study is thus unquestionable. Further, when a researcher asks service users their opinion of a questionnaire that is popular in psychological research, and they respond that it is invasive and irrelevant to their lives, then it becomes difficult for the researcher to use that instrument. This is not because researchers cannot ignore service users’ views, but rather because it forcefully exposes the gap in relevance between mainstream research and the interests and lives of service users. While mental health activists have argued this viewpoint for many years [1], experiencing it first hand is persuasive.

Our study illustrates a participatory approach to iteratively incorporating experiential and research-based knowledge into system design and evaluation. Participatory approaches like CBPR are expected to speed translation of knowledge into practice, along with reducing health disparities [5,9]. To strengthen this type of approach, scientific communities need to share not only the outcomes of system use, but also the rationales for decisions made in research and service design processes. If it is clear why a certain decision has been made, it is easier to incorporate the crucial aspects into rapidly developing new technologies, as well as identify how new technologies might be responsibly put to use. Decisions are not just based on research, but also on values and cultural contexts of participants. This is an important justification for service user involvement and approaches such as CBPR. Increased skills in these approaches should better enable us to exploit new technologies in ethical, timely and culturally relevant ways.

## Conclusion

Starting down the path of involving service users in intervention design fosters commitment to follow through also in the implementation and research phases. While this work can be time-consuming for researchers, and give little return in terms of prestige, it is probably vital to increasing the likelihood of success of person-centered service innovations.

## Competing interests

The authors declare that they have no competing interests.

## Author information

DG is a PhD, psychologist and senior scientist with 20 years’ experience heading telemedicine and ehealth research and development projects. Her focus has been on person-centered systems for different health conditions, including mental health.

MS has several years’ experience as a psychiatric nurse, is now a PhD fellow at Oslo University Hospital as well as a consultant at the Department of Psychiatry Blakstad, Division of Mental Health and Addiction, Vestre Viken Hospital Trust.

LSE is among the first service users in Norway to have completed a training program that qualifies as a mental health service user consultant. For many years she has been voicing the experiences and concerns of service users in Norway and is now completing a newly developed course for co-researchers in mental health.

## Authors’ contributions

All authors participated in the design of the study, funding acquisition, and conceptualization of the case report. DG made a first draft, MS and LSE made substantial edits. All authors read and approved the final manuscript.
